# Effectiveness of Foliar Biofortification of Carrot With Iodine and Selenium in a Field Condition

**DOI:** 10.3389/fpls.2021.656283

**Published:** 2021-05-21

**Authors:** Roksana Rakoczy-Lelek, Sylwester Smoleń, Marlena Grzanka, Krzysztof Ambroziak, Joanna Pitala, Łukasz Skoczylas, Marta Liszka-Skoczylas, Hubert Kardasz

**Affiliations:** ^1^Intermag Sp. z o.o., Olkusz, Poland; ^2^Department of Plant Biology and Biotechnology, Faculty of Biotechnology and Horticulture, University of Agriculture in Kraków, Kraków, Poland; ^3^Laboratory of Mass Spectrometry, Faculty of Biotechnology and Horticulture, University of Agriculture in Kraków, Kraków, Poland; ^4^Department of Plant Product Technology and Nutrition Hygiene, Faculty of Food Technology, University of Agriculture in Kraków, Kraków, Poland; ^5^Department of Engineering and Machinery for Food Industry, Faculty of Food Technology, University of Agriculture in Kraków, Kraków, Poland

**Keywords:** selenium, iodine, biofortification, carrot, foliar application dates

## Abstract

Iodine (I) and selenium (Se) are essential to human and animal development. There is a worldwide deficit of I and Se in the diet of humans, as well as in animals. It is advisable to enrich plants with these elements to ensure adequate uptake in animals and humans. The aim of this study was to determine the efficacy of the application of I and Se in the cultivation of carrot crops, to better understand the metabolic pathways and processes of I applied through foliar spray. Carrots were fertilized with 4-fold foliar applications of I and Se, which were applied as the liquid fertilizers “I + Se”, “Solo iodine” and “Solo selenium”, all containing an organic stabilizer, in two field trials. Foliar nutrient applications of I and Se were translocated by the plant for storage in the roots. The level of enriched I and Se in the roots was considered safe for the consumer. The Recommended Daily Allowance values for I and Se in the roots of 100 g of fresh carrots are 4.16% and 4.37%, respectively. Furthermore, I and Se accumulated in the roots to a level that was physiologically tolerated by carrot. Biofortification through foliar feeding did not impact negatively on the yield or quality of the carrot crop. Iodides applied via foliar application were the dominant form of I in the plant tissues and were included in the metabolic process of the synthesis of iodosalicylates, iodobenzoates, iodotyrosine (I-Tyr), and plant-derived thyroid hormone analogs. No synergistic or antagonistic interaction between I and Se, with respect to the effectiveness of biofortification in roots, was observed in any treatments. The molar ratio of I:Se in the roots after foliar application of both elements was approximately 1.6:1 and was similar to the control (1.35:1).

## Introduction

Iodine (I) and selenium (Se) are micronutrients that are essential for proper functioning of humans and animals (Gonzali et al., 2017; Márquez et al., 2020). Both of these microelements are required for the optimal synthesis of the thyroid hormones, thyroxine (T4) and triiodothyronine (T3) (Zimmermann, 2011; Mehdi et al., 2013). I deficiency causes severe health problems that affect approximately 1.5 billion people worldwide (White and Broadley, 2005; Blasco et al., 2012; Choudhry and Nasrullah, 2018). Roughly 15% of the global population is afflicted with diseases caused by Se deficiency, and 500 million to 1 billion people are exposed to a Se-deficient diet (Athar et al., 2020; Márquez et al., 2020). The Recommended Daily Allowance (RDA) for I and Se depends on age and gender and amounts to 90–250 μg I per day (Gonzali et al., 2017) and 60–70 μg Se per day (Mehdi et al., 2013).

I and Se are considered beneficial plant elements. However, they are not classified as essential for the metabolism of vascular plants (Pilon-Smits et al., 2009; Gonzali et al., 2017). Se is the only essential micronutrient for plants classified as a hyperaccumulator of Se (Pilon-Smits et al., 2009). This positive effect of Se was confirmed in studies on ryegrass, tomato, and lettuce, which are crop plants that are not Se hyperaccumulators (Hartikainen, 2005; Pilon-Smits et al., 2009). In contrast to I, Se is effective in stimulating growth and plant development (Xue et al., 2001; Hartikainen, 2005). Se protects cell membranes against lipid peroxidation influences on tocopherol levels (Xue et al., 2001; Hartikainen, 2005), increases the activity of glutathione peroxidase (GPX) (Xue et al., 2001; Hartikainen, 2005; Pilon-Smits et al., 2009), and increases resistance to ultraviolet light (Mostafa and Hassan, 2015). Indian mustard (*Brassica juncea*) plants grown with Se showed repellent properties against herbivory by caterpillars (*Pieris rapae*) (Hanson et al., 2003).

Both inorganic and organic forms of Se are taken up by plants (Sors et al., 2005; Winkel et al., 2015; Smoleń et al., 2016b). The inorganic form of Se is selenate (SeO_4_^2–^), which is effectively taken up by the roots, transported through the phloem and xylem, and accumulated in shoots and leaves (Winkel et al., 2015). Selenate levels are reduced after being absorbed by the leaves, and Se is involved in protein metabolism, resulting in the production of selenoproteins (Sors et al., 2005; Mehdi et al., 2013). Selenate and its organic combinations are bioavailable forms and are actively taken up via the sulfate pathway by sulfate transporters (Sors et al., 2005; Pilon-Smits et al., 2009; White and Broadley, 2009).

The uptake of I from the soil is not as effective as in the case of Se. I is present in the soil in the form of inorganic salts, such as iodide (I^–^), iodate (IO_3_^–^), and organic molecules (White and Broadley, 2009). The availability and mobility of I in the soil depend on the soil conditions and its physicochemical properties (Amachi, 2008). In most of the types of soil, I is not available for the plants. Soils that are fertile and rich in organic matter (humus) have a significant effect on limiting the bioavailability of inorganic I present in the soil (White and Broadley, 2005; Dai et al., 2009). Acidic soils containing aluminum and iron ions also reduce the bioavailability of I. Mainly soils with a neutral pH provide the best conditions for I mobility. The forms in which I occurs in the soil depend on the soil conditions. Iodides dominate in low pH and redox conditions, whereas iodates are common in oxidative conditions and soils with a neutral to alkaline pH (White and Broadley, 2009; Blasco et al., 2012).

Another important limiting factor in the enrichment of plants with I is low I mobility in the phloem that results in difficulties in the effective enriching of cereals, rice, and maize and low I accumulation in grain (Blasco et al., 2012). The most effective accumulation of I is observed when I is transported by xylem. Therefore, enrichment with I is most effective in leafy vegetables (Mackowiak and Grossl, 1999; Blasco et al., 2012).

Generally, the uptake of I by the roots from the soil and transport to the generative plants organs is limited. Therefore, various agrotechnical methods for biofortification with I and Se of crops have been developed. One of these methods is foliar application, which is more effective than soil fertilization.

Signore et al. (2018) showed that foliar application of high doses of I (500 mg ⋅ L^–1^) did not have phytotoxic effects on the leaves of carrots cultivated in the field. Foliar fertilization with potassium iodate (KIO_3_) (at a high dose of 500 mg ⋅ L^–1^) of carrots cultivated in a greenhouse did not show the significant I enrichment effect seen in foliar application of the same dose in carrots cultivated in the field. However, the addition of KIO_3_ in lower doses (50 mg ⋅ L^–3^) to the nutrient solution resulted in significant enrichment of I in carrot roots, as well as in visible phytotoxic properties present on the leaves.

Research in a field of biofortification of plants with simultaneous applications of I and Se has been undertaken in several crops such as lettuce (Sahin, 2020), buckwheat and pumpkin sprouts (Germ et al., 2015), kohlrabi (Golob et al., 2020), and Indian mustard (Golubkina et al., 2018). The experiments were carried out in pots, using hydroponic systems under field conditions. Most of the research was focused on enriching plants with I and Se through soil fertilization or hydroponic system with a nutrient solution. There was no effect on the uptake and accumulation of these elements by plants, based on the simultaneous application of I and Se (Zhu et al., 2004; Sahin, 2020).

Foliar biofortification of cereals (including wheat and rice) with I and Se has been widely studied (Zou et al., 2019; Cakmak et al., 2020). Protocols for carrot biofortification through soil fertilization with I and Se have been also compiled (Smoleń et al., 2016a, 2019). However, little is known about carrot enrichment with I and Se via foliar application. Additionally, the organic I metabolites that may be present in the leaves and roots of the carrot plant have not yet been determined.

In this article, the efficacy of carrot biofortification with I and Se through foliar application has been studied.

This study was aimed at determining the effect of the foliar application of both I and Se on the biofortification efficiency in the roots of the carrot plant. The efficacy of I and Se accumulation was assessed in the carrot plant after the foliar application of the formulations “Solo iodine” containing only I, “Solo selenium” containing only Se, and the newly developed fertilizer “I + Se”. Hypothesis whether I and Se could be uptaken by the leaves and effectively transported to the storage root has been investigated. The extent to which organic forms of I, such as iodosalicylates, iodobenzoates, and iodotyrosine (I-Tyr), as well as plant-derived thyroid hormone analogs (PDTHAs), such as T3 and T4, were metabolized in the carrot after foliar application of I and Se was investigated.

## Plant Material and Treatments

### Study Design

Two field trials were undertaken in 2019 and 2020, respectively, for the carrot (*Daucus carota* L.) cultivar “Octavo F_1_”. The carrots were cultivated on a farm specializing in the cultivation of root vegetables in southeastern Poland. This carrot variety is harvested in autumn for the fresh market. Wheat was the forecrop each year, and carrots were cultivated in a soil with high clay content (Table 1). The soil was characterized by a low electrical conductivity (EC) and low levels of mineral nitrogen (N) before cultivation. The soil had low potassium (K) levels and optimal magnesium (Mg) levels for cultivation of carrots over both years. In 2020, the content of calcium (Ca) and organic matter was naturally higher than in 2019. Each year, NPK was added as a fertilizer to the soil before sowing (applied at levels of 100 mg N ⋅ dm^–3^, 80 mg phosphorus (P) ⋅ dm^–3^, 200 mg K ⋅ dm^–3^), to supplement the levels of N, P, and K, which are essential elements for optimal growth of carrots (Sady, 2006). Commercially available fertilizers were used, which comprised 50% of K as potassium sulfate (Siarkopol Tarnobrzeg, Poland) and 50% of K as potassium chloride (Zakład Obrotu Towarami Sp. z o.o., Dwikozy, Poland), Polifoska 5 (Grupa Azoty, Zakłady Chemiczne Police SA, Poland), and the N fertilizer Sulfamo, with 23% N (Timac Agro, Roullier Group, France). Chemical plant protection against weeds, diseases, and pests was applied in accordance with the current plant protection program in Poland. Carrots were sowed in raised beds in single rows, 40 cm wide and 30 cm high, at a seeding rate of approximately 1.6 million seeds per hectare. The seeds were sown on April 30, 2019, and April 15, 2020.

**TABLE 1:** The chemical properties of the soil prior to carrot cultivation.

Physicochemical soil characteristic	Year 2019	Year 2020
pH_(H2O)_	6.48	7.50
EC (mS ⋅ cm^–1^)	0.48	0.14
Macroelements:		
N-NH_4_ (mg ⋅ dm^–3^)	6.0	0.29
N-NO_3_ (mg ⋅ dm^–3^)	3.0	8.81
N-NH_4_ + N-NO_3_ (mg ⋅ dm^–3^)	9.0	9.0
P (mg ⋅ dm^–3^)	60.0	33.2
K (mg ⋅ dm^–3^)	134.0	136.9
Mg (mg ⋅ dm^–3^)	70.0	105.5
Ca (mg ⋅ dm^–3^)	1,520.0	4,585.2
Soil organic matter (%)	0.80	1.72
Type of soil	Heavy soil	Heavy soil

The study included foliar biofortification of carrots with I and Se (applied together and separately), using the newly developed water-soluble liquid concentrate fertilizers “I + Se”, “Solo iodine” and “Solo selenium” (Table 2). An untreated crop was included as the control in the experiments. The methods for conducting the experiment are described in Table 2. Four foliar applications of fertilizers were applied to the carrot crop at the following doses: 200 g I + 10 g Se ⋅ ha^–1^, 400 g I + 20 g Se ⋅ ha^–1^, 200 g I ⋅ ha^–1^, 400 g I ⋅ ha^–1^, 5 g Se ⋅ ha^–1^, and 10 g Se ⋅ ha^–1^. The field trials were conducted in a split-plot design with four repetitions per treatment and a plot size of 10 m^2^. Foliar fertilization was applied in the morning during fine weather.

**TABLE 2:** Experimental treatments of foliar application (foliar biofortification) of carrot with iodine and selenium–the methodological information.

Treatments of foliar application: the total dose of I and/or Se in four applications	Type of fertilizer^1^ and way dosage of I and Se
Control	Without fertilizer–spray only with water
200 g I + 10 g Se ⋅ ha^–1^	0.25 dm^3^ “I + Se^2^” ⋅ ha^–1^ per one application × four applications = 1.0 dm^3^ “I + Se” ⋅ ha^–1^ “I + Se” liquid fertilizer containing iodine and selenium in the form I^–^ and SeO_4_^2–^ with organic stabilizer/enhancer^®^. Concentration of iodine and selenium in “I + Se”, respectively, 15.7% wt/vol and 0.79% wt/vol Molar mass ratio of I:Se in the fertilizer = 12.4:1
400 g I + 20 g Se ⋅ ha^–1^	0.5 dm^3^ “I + Se” ⋅ ha^–1^ per one application × four applications = 2.0 dm^3^ “I + Se” ⋅ ha^–1^. Other information as above
200 g I ⋅ ha^–1^	0.25 dm^3^ “Solo iodine^2^” ⋅ ha^–1^ per one application × four applications = 1.0 dm^3^ “Solo iodine” ⋅ ha^–1^ “Solo iodine”: liquid iodine fertilizer in form I^–^ with organic stabilizer/enhancer Concentration of iodine 16.6% wt/vol
400 g I ⋅ ha^–1^	0.5 dm^3^ “Solo iodine” ⋅ ha^–1^ per one application × four applications = 2.0 dm^3^ “Solo iodine” ⋅ ha^–1^ Other information as above
5 g Se ⋅ ha^–1^	0.125 dm^3^ “Solo selenium^2^” ⋅ ha^–1^ per one application × four applications = 0.5 dm^3^ “Solo selenium” ⋅ ha^–1^ “Solo selenium^2^”: liquid selenium fertilizer in form SeO_4_^2–^ with organic stabilizer/enhancer Concentration of selenium 0.95% wt/vol
10 g Se ⋅ ha^–1^	0.25 dm^3^ “Solo selenium^2^” ⋅ ha^–1^ per one application × four applications = 1.0 dm^3^ “Solo selenium” ⋅ ha^–1^ Other information as above
**Schedule and method of foliar application for each treatment of foliar application**	
Quantity and dates (growth phase) of foliar application in 2019 and 2020	1st BBCH 16–first decade of July 2nd BBCH 21–end of July 3rd BBCH 35–mid August 4th BBCH 42–end of August
The amount of working solutions	500 dm^–3^ ⋅ ha^–1^–tap water was used in the control and tested combinations
Sprayer type	Foliar treatments on plots performed with a manual, pneumatic pressure sprayer
Date of completion of the field experiment—carrot harvest	First half of September of 2019 and 2020; BBCH 46-48 phase. Carrot harvest, yield evaluation and sampling of leaves and roots for chemical analysis

*1: Surfactants ADITENS MAX (Intermag Sp. z o.o., Olkusz, Poland) were used in the control and in each treatment with the foliar application of iodine and/or selenium.*

*2: “I + Se”, “Solo iodine” and “Solo selenium”–experimental liquid fertilizers for foliar feeding developed in the project entitled “Modern agrochemical preparations based on biodegradable ligands and other natural compounds stimulating resistance, enabling plant biofortification, intended to be used as a part of Integrated Plant Production,” which was co-financed by the European Union from the European Regional Development Fund under Smart Growth Operational Program 2014–2020. Project implemented as part of the competition of the National Centre for Research and Development: Szybka Ścieżka, project no. POIR.01.01.01-00-0024/15, implemented by Intermag Sp. z o.o., Olkusz, Poland. Fertilizers at the registration stage. Fertilizers are stable and maintain the initial content of I and/or Se during storage over 2 years’ time in a tightly closed containers.*

*Organic stabilizer/enhancer^®^ (the same stabilizer for iodine and selenium) is proprietary, the know-how of the producer of these fertilizers, i.e., Intermag Sp. z o.o., Olkusz, Poland. Stabilizer/enhancer^®^ of I and Se is based on a mixture of organic compounds occurring exogenously in the plants.*

In the study, 5 g I and 10 g Se ⋅ ha^–1^ were tested using an application of “Solo selenium” (Se-alone fertilizer). Doses of 10 g and 20 g Se ⋅ ha^–1^ were used for the combined application of I and Se, whereas the 5 g Se ⋅ ha^–1^ dose was not tested. In screening test carried out in the greenhouse on carrot plants, it was observed that the foliar application of Se in doses of 10 and 20 g of Se combined with I had a similar effect on Se accumulation in plants as the foliar application of Se in doses of 5 and 10 g Se ⋅ ha^–1^ (the obtained results are presented in the Supplementary Table 1). Field trials were conducted under commercial conditions at the agricultural research farm over 2 years to determine the effects on carrot plants.

The fertilizers used in the field trials were developed based on the research project called “Modern agrochemical preparations based on biodegradable ligands and other natural compounds stimulating resistance, enabling plant biofortification, intended to be used as a part of Integrated Plant Production”, which was co-financed by the European Union via the European Regional Development Fund under the Smart Growth Operational Program 2014–2020. The project was implemented under the National Centre for Research and Development: Szybka Ścieżka, project no. POIR.01.01.01-00-0024/15 (Intermag Sp. z o.o., Olkusz, Poland). The studies described in the publication were preceded by the formulation of an appropriate concentration of I and Se in liquid fertilizers, with addition of an organic stabilizer/enhancer^®^ (biodegradable stabilizer). The research and development for the formulation consisted of screening test on carrot and the determination of safe concentrations of I and Se in working solution for plants. The results of the test made it possible to develop a protocol of foliar biofortification of carrot plants with I and Se, which detail the safe dosage per hectare, required concentrations of I and Se in working solution, and recommended application dates. This study describes the final stage of research and testing for the efficacy of fertilizers for carrot biofortification (enrichment) with I and Se.

The total yield of leaves and roots harvested, as well as the average weight of roots and leaves from single plants, was measured (Table 2). Roots were sorted into marketable and non-marketable yields, according to the criteria described by Smoleń et al. (2019). At harvest, samples of approximately 5 kg of carrot roots of marketable yield were chosen from each plot for laboratory analysis (allowing for repetition). Samples of healthy, fully developed leaves with a mass of approximately 0.2 kg were collected from each replicate.

### Plant Analyses

The leaves and roots were washed in tap water before analysis. The samples of fresh roots and leaves were then homogenized. The dry weight content in these samples was determined using the oven-drying method, at 105°C. The content of total carotenoids in the fresh homogenized roots, extracted with acetone/n-hexane (4:6), was determined using spectrophotometry. Total dissolved solids (% Brix) in roots were measured using the Atago Palette PR-32, a digital refractometer. The analyses were performed using the methods described by Smoleń et al. (2014b, 2019).

Total sugars, as a sum of glucose, fructose, and sucrose, were measured in the ethanol extracts, using the reverse capillary electrophoresis technique with the PA 800 Plus system (Beckman Coulter, United States). Capillaries of ø 50 μm and total length of 60 cm (10 cm for detection) were used. A positive power supply of 15 kV was applied, and the temperature was set at 25°C. The running buffer solution comprised 20.0 mmol/L sorbic acid, 0.20 mmol/L CTAB, and 40 mmol/L NaOH, pH 12.2 (Rizelio et al., 2012).

In addition, approximately 400 g (fresh weight) of a homogenized sample of leaves and roots was frozen at −20°C and then dried by lyophilization. A freeze dryer was used, namely, the PG90-Lx4/14 type LIOex-4/330-A4 (ARTVAC-Plus, Mȩcina, Krzeszowice, Poland). After drying, the samples were ground in a laboratory mill using a Pulverisette 14 Fritsch (Idar-Oberstein, Germany) variable speed rotor mill, with a 0.5-mm sieve. The Se and I content of the dried leaves and roots was analyzed with an inductively coupled plasma–tandem mass spectrometry (ICP-MS/MS) triple quadruple spectrometer (iCAP TQ ICP-MS Thermo Fisher Scientific, Bremen, Germany). The Se content was determined after microwave mineralization. Samples (0.1 g) were placed into 55 mL TFM vessels and were mineralized in 10 mL 65% super pure HNO_3_ (Merck no. 100443.2500), in a Mars 5 Xpress (CEM, United States) microwave digestion system (Kalisz et al., 2019). The I content was determined after extraction using tetramethylammonium hydroxide (TMAH) (Sigma–Aldrich Co., LLC, St. Louis, MO, United States), according to the Polish Standard-European Standard procedure (European Standard Norme Européenne Europäische Norm, 2008) with modifications described by Smoleń et al. (2019).

Speciation of I, i.e., iodides and iodates, was analyzed in the dried samples of roots and leaves, using high-performance liquid chromatography (HPLC)–ICP-MS/MS. The content of these two I ions was measured using a modified extraction procedure described by Smoleń et al. (2016c), whereby 0.05 g of air-dried, ground plant samples were mixed with an extraction solution containing 4 cm^3^ of 25% TMAH (Sigma–Aldrich Co., LLC) and 10 cm^3^ 0.1 M NaOH (Chempur, Piekary Śla̧skie, Poland), in 1 dm^3^ of demineralized water. The samples were placed in 7-mL polypropylene tubes, whereupon 5 mL of the extraction mixture was added. Once mixed, the samples were incubated for 1 h at 50°C in an ultrasonic bath and then cooled to approximately 20°C, mixed thoroughly, and centrifuged for 15 min at 4,500 revolutions/min. The supernatants were filtered through a 0.22-μm syringe filter. The content of I ions in filtered samples was analyzed using HPLC–ICP-MS/MS. For I^–^ and IO_3_^–^ speciation forms, HPLC (Thermo Scientific Ultimate 3000; Thermo Fisher Scientific, Bremen, Germany) was coupled to ICP-MS/MS (iCAP TQ). This method employed a strong anion exchange column (Thermo Scientific; Dionex IonPac AS11 [4 × 250 mm]) and a pre-column (Thermo Scientific; Dionex IonPac AG11 [4 × 50 mm]). The column temperature was set to 30°C. Demineralized water, 50 mM NaOH, and 0.5% TMAH were used as eluents. To separate both I ions, a mobile phase was used, containing 2.5 mM NaOH and 0.125% TMAH with an isocratic flow. The flow rate was 1.5 mL/min, with an injection volume of 10 μL, and total analysis time of 7 min. The 127I.16O isotope of I was determined, using the S-TQ-O2 mode. Standards were prepared through dissolution of KI and KIO_3_ (Sigma–Aldrich Co., LLC) in demineralized water.

Leaves and roots were analyzed for salicylic acid (SA), benzoic acid (BeA), iodosalicylates [5-iodosalicylic acid (5-ISA) and 3,5-diiodosalicylic acid (3,5-diISA)], iodobenzoates [2-iodobenzoic acid (2-IBeA), 4-iodobenzoic acid (4-IBeA), 2,3,5-triiodobenzoic acid (2,3,5-triIBeA)], I-Tyr, and PDTHA such as T3 and T4, using the liquid chromatography (LC)–MS/MS system after extraction with 75% ethanol (Smoleń et al., 2020). Measurements were made using the HPLC Ultimate 3000 system (Thermo Scientific) and an LC-MS/MS: 4500 Qtrap, Sciex spectrometer. Chromatographic separation was carried out on a Luna 3 μm phenyl-hexyl 100 Å (150 × 3 mm, internal diameter 3 μm) column (Phenomenex, Torrance, CA, United States). Electrospray ionization in negative ion mode was used. MS/MS was performed for quantitative analysis. The transitions monitored for SA, BeA, 5-ISA, 3,5-diISA, 2-IBeA, 4-IBeA, 2,3,5-triIBeA, I-Tyr, TransThy-T3/T4trans (T3 and T4 proteins), and for SA-d4 were 136.8/93.1, 120.9/76.9, 262.9/126.7, 388.8/126.7, 246.9/126.6, 246.9/144.7, 498.7/454.4, 306.1/126.8, 649.9/632.7, 775.7/574.6, and 141/96.8, respectively. The LC-MS/MS system was controlled using Analyst 1.7 with HotFix 3 software, which was also used for data processing. Analysis of the content of SA and BeA, I derivatives of these acids, and PDTHA in leaves and roots was performed for three selected treatments, i.e., the control and foliar application of “I + Se” (200 g I + 10 g Se ⋅ ha^–1^) and “Solo iodine” (200 g I ⋅ ha^–1^). These treatments were selected to determine the preferred pathways of I metabolism in plants after foliar application of this element. In addition, these treatments were selected to determine whether, and to what extent, the additional application of Se affects metabolism of I in leaves and roots.

The transmittance method in the CIE*Lab* system was used to measure the color of roots of carrot plants (Wrolstad and Smith, 2017), using a Konica Minolta CM-3500d spectrophotometer (Konica Minolta Sensing, Osaka, Japan). This system used the reflectance method, with the illuminant D65 in a Petri dish (6 cm in diameter and 4 cm in height), at an observer angle of 10°. This allowed for measurement of values for the parameters of *L*^∗^ (lightness), *a*^∗^ (redness), and *b*^∗^ (yellowness). The indices Δ*E*^∗^ (total color difference), Δ*C*^∗^ (total saturation difference), and Δ*H*^∗^ (total hue difference) were also calculated.

Color changes between the biofortified and unfortified samples of carrot roots were expressed as the total color difference (Δ*E*^∗^). This parameter was calculated as the Euclidean distance between two points in the three-dimensional space determined by *L*^∗^, *a*^∗^, and *b*^∗^ using the following formula:

Δ⁢Eab*=Δ⁢a2*+Δ⁢b2*+Δ⁢L2*

where Δ*a*^∗^, Δ*b*^∗^, and Δ*L*^∗^ are the differences between the reference and analyzed sample. The quantitative attribute of color is Chroma (*C*^∗^), which was calculated using following equation:

C*=a2*+b2*

whereas Δ*C*^∗^ is the difference between the *C*^∗^ parameter value obtained for the analyzed sample and the reference.

Total hue difference (Δ*H*^∗^) was determined according to the following formula:

$$\text{Δ}\,H^{{}^{*}}=\sqrt{{\left(\text{Δ}\,E^{{}^{*}}\right)}^{2}-{\left(\text{Δ}\,L^{{}^{*}}\right)}^{2}-{\left(\text{Δ}\,C^{{}^{*}}\right)}^{2}}$$

that calculated the indices of color, averaged over 20 measurements for each replication of each experimental treatment.

The results of the color parameters were interpreted in accordance with the criteria established by the International Commission on Illumination (CIE) (Hutchings, 1999).

Eight individual soil samples were randomly collected from the study area. Soil samples were taken from the 0- to 30-cm layer, to allow for characterization of the chemical properties of the soil prior to the start of the study (Table 1). The description of these results is presented in section “Results”.

The following procedures were used for soil samples analyses: pH was measured potentiometrically in soil samples mixed with water (1:2 vol/vol, soil:H_2_O), and the EC was analyzed using a conductivity meter. After extraction with 0.03 M acetic acid, the concentrations of P, K, Mg, and Ca were determined with the ICP-OES technique (Nowosielski, 1988). The extracts were also used to determine the concentrations of N minerals in the form of N-NH_4_ and N-NO_3_ using the AQ2 discrete analyzer (SEAL Analytical, Mequon, WI, United States), based on the protocol developed by the manufacturer. The organic matter in the soil was determined using the Tiurin method (Nowosielski, 1988; Komornicki et al., 1991).

### Meteorological Data

The average daily temperature from April to September in 2019 and 2020 was similar. In the 2019, the warmest month was June, and in 2020, it was August (Table 3). The largest difference in the average daily temperature between 2019 and 2020 was in June. Rainfall from April to September in the 2019 was higher by 64.8 mm (17%), compared to 2020. In the 2020, rainfall was evenly distributed from April to September. In 2019, June had the lowest average rainfall (14.5 mm), and May and August had the highest rainfall, with 131.7 and 128.7 mm, respectively.

**TABLE 3:** Meteorological data for the carrot cultivation period in 2019 and 2020.

Month	Monthly mean daily air temperature (°C)	
	Year 2019	Year 2020
April	8.49	6.08
May	11.44	10.25
June	20.33	17.09
July	18.54	18.19
August	17.27	19.42
September	13.69	14.47
Mean	14.96	14.25

	**Monthly sum of rainfall (mm)**	

April	59.1	23.0
May	131.7	76.2
June	14.5	71.8
July	22.0	55.7
August	128.7	82.9
September	78.4	60.0
Sum	434.4	369.6

### Biofortification Targets Based on Consumer Safety for I and Se Enriched Carrots

The percentage of the RDA for I (RDA-I) and Se (RDA-Se) in 100-g portions of the roots of fresh carrots was calculated, based on chemical analyses of the roots. In addition, the molar ratio of I:Se in fresh carrot roots was calculated, dependent on the foliar application of I, as well as the hazard quotient (HQ). The intake of I and Se was calculated based on the consumption of 100 g of fresh carrot roots by adults (average of 70-kg body weight). All of the calculations were based on the methods described by Smoleń et al. (2019).

### Data Analysis

TIBCO Software Inc. (2017). Statistica (data analysis software system), version 13.3 PL https://www.statsoft.pl/, was used for analysis of variance (ANOVA) by ANOVA module. The Tukey test was used for determining the significance between the means at *p* < 0.05.

## Results

### Carrot Yield

No statistically significant effects of foliar applications of I, Se, and I + Se, applied in two doses, were observed for the yield of leaves and roots, the marketable yield as a percentage of the total yield, or the average weight of roots and leaves from one plant, compared to the control (Table 4).

**TABLE 4:** Yield of leaves and storage roots, share of the marketable yield in the total yield, and average weight of single storage roots and leaves from one plant, depending on the foliar application of iodine and selenium.

Foliar application	Yield of carrot leaves (t ⋅ ha^–^^1^)	Total yield of carrot storage roots (t ⋅ ha^–^^1^)	Marketable yield of carrot roots (t ⋅ ha^–^^1^)	Non-marketable yield of carrot roots (t ⋅ ha^–^^1^)
Control	39.6 ± 8.68a	100.4 ± 8.18a	85.2 ± 4.25a	15.2 ± 4.48a
200 g I + 10 g Se	37.5 ± 7.00a	95.5 ± 4.78a	81.5 ± 2.87a	14.0 ± 4.23a
400 g I + 20 g Se	38.2 ± 7.67a	100.2 ± 6.21a	84.2 ± 3.31a	16.0 ± 4.27a
200 g I	34.6 ± 6.56a	100.2 ± 7.51a	83.8 ± 3.09a	16.4 ± 4.63a
400 g I	38.1 ± 7.33a	106.3 ± 10.22a	93.8 ± 7.02a	12.5 ± 4.45a
5 g Se	37.8 ± 7.83a	101.6 ± 9.17a	85.9 ± 6.59a	15.7 ± 5.70a
10 g Se	34.0 ± 5.55a	97.1 ± 6.76a	79.4 ± 4.47a	17.7 ± 4.20a
Test *F* for foliar application	n.s.	n.s.	n.s.	n.s.
Test *F* for foliar application × year of study	n.s.	n.s.	n.s.	n.s.

	**Share of the marketable yield in the total yield of storage roots (%)**	**Total biological yield (roots + leaves) (t ⋅ ha**^–^**^1^)**	**Average weight of single storage roots (g ⋅ plants**^–^**^1^)**	**Average weight of leaves from one plant (g ⋅ plants**^–^**^1^)**

Control	86.7 ± 3.46a	140.0 ± 16.53a	63.9 ± 3.97a	27.0 ± 1.64a
200 g I + 10 g Se	86.3 ± 3.70a	133.0 ± 11.25a	63.8 ± 3.58a	25.0 ± 0.28a
400 g I + 20 g Se	85.2 ± 3.50a	138.4 ± 13.44a	66.7 ± 4.14a	24.0 ± 1.25a
200 g I	85.3 ± 3.24a	134.8 ± 13.43a	67.3 ± 6.49a	23.6 ± 1.14a
400 g I	89.8 ± 2.93a	144.4 ± 17.35a	58.6 ± 3.07a	23.6 ± 0.63a
5 g Se	86.1 ± 4.87a	139.4 ± 16.01a	63.6 ± 3.29a	23.0 ± 0.67a
10 g Se	82.9 ± 3.50a	131.1 ± 12.04a	67.2 ± 7.55a	24.4 ± 1.35a
Test *F* for foliar application	n.s.	n.s.	n.s.	n.s.
Test *F* for foliar application × year of study	n.s.	n.s.	n.s.	n.s.

*Application 200 g I + 10 g Se ⋅ ha^–1^ and 400 g I + 20 g Se ⋅ ha^–1^ by “I + Se” fertilizer, application of 200 g and 400 I ⋅ ha^–1^ by “Solo iodine” fertilizer, and application 5 and 10 g Se ⋅ ha^–1^ by “Solo selenium”⋅fertilizer. Means followed by the same letters are not significantly different for *p* < 0.05. ±, standard error (*n* = 8); n.s., not significant for *p* < 0.05. Average for the years 2019–2020.*

### Content of I and Se in the Carrot Plants and Consumer Safety

**FIGURE 1:**
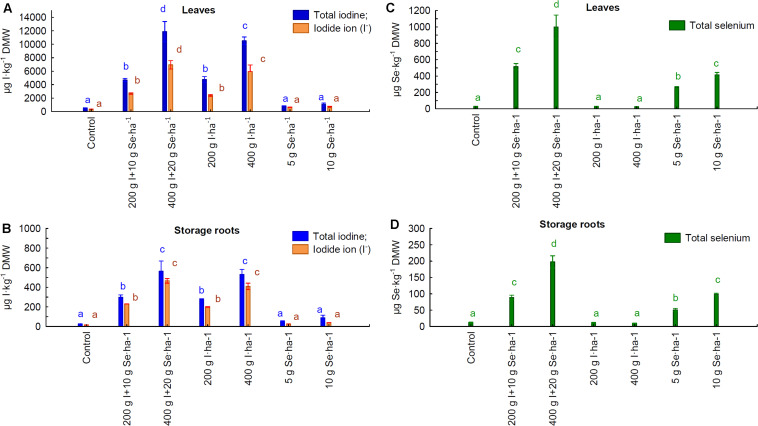
Total iodine, iodide ion (I^–^ ) **(A,B)**, and total selenium **(C,D)** contents in carrot leaves **(A,C)** and storage roots **(B,D)**, depending on the foliar application of both elements. Application 200 g I + 10 g Se ⋅ ha^–1^ and 400 g I + 20 g Se ⋅ ha^–1^ by “I + Se” fertilizer, application of 200 g and 400 I ⋅ ha^–1^ by “Solo iodine” fertilizer, and application 5 g and 10 g Se ⋅ ha^–1^ by “Solo selenium”⋅fertilizer. The results of the test *F* for “foliar application” for all qualitative features in panels **(A–D)** were statistically significant. The results of the test *F* for “foliar application × year of study” for all qualitative features in panels **(A–D)** were not statistically significant. Means followed by the same letters are not significantly different for *p* < 0.05; bars indicate standard error (*n* = 8). Average for the years 2019–2020.

Foliar application of the I fertilizer “Solo iodine” and the I-Se fertilizer “I + Se” resulted in a significant increase in the total content of I (I-total) and of iodide (I^–^) ions in the leaves and roots of carrot plants (Figure 1). When a higher I dose of 400 g I ⋅ ha^–1^, rather than 200 g I ⋅ ha^–1^, of both fertilizers was applied, there was a proportional increase in the accumulation of I-total and I^–^ in the leaves and roots. Similar increases in I-total and I^–^ accumulation were observed in the roots after the application of the same doses of I (200 and 400 g I ⋅ ha^–1^, respectively) with the “I + Se” and “Solo iodine” fertilizers. A significant increase in the accumulation of I-total and I^–^ was observed in the leaves after application of “I + Se” (400 g I ⋅ ha^–1^), which was approximately 17% greater than was observed after the application of “Solo iodine”. This was not the case when doses of 200 g I ⋅ ha^–1^ were applied. The levels of I-total in all foliar applications of “I + Se” and “Solo iodine” were 10–20 times higher in the leaves than in the roots.

There was a significant increase in the levels of Se in the leaves and roots compared to the control, after the plants were sprayed with the “I + Se” and “Solo selenium” fertilizers (Figure 1). The Se content in leaves and roots increased proportionally to increasing concentrations of Se (5 and 10 g of Se ⋅ ha^–1^), applied as the fertilizer “Solo selenium”. Application of 10 g Se ⋅ ha^–1^ with “Solo selenium” and “I + Se” had similar effectiveness in enriching the roots and leaves with Se. The use of the “I + Se” at a dosage of 20 g Se ⋅ ha^–1^, compared with 10 g Se ⋅ ha^–1^, resulted in a 2-fold increase in the levels of Se in leaves and roots. The levels of Se in the roots were approximately 4.5-fold lower than in the leaves in plants treated with the foliar fertilizers “Solo selenium” and “I + Se”.

Foliar applications of “Solo selenium” and “Solo iodine” did not show any significant changes in the levels of I and Se in leaves and roots, respectively, when compared to the control (Figure 1).

Consumption of 100 g of fresh carrot roots from plants enriched with I from the fertilizer “I + Se” could provide between 1.96 and 4.19% of RDA-I and 1.98 and 4.37% of RDA-Se for the lower and higher doses of the fertilizer, respectively (Table 5). Percentages of RDA-I and RDA-Se from plants enriched with the “Solo iodine” and “Solo selenium” fertilizers were similar to the equivalent doses of I and Se applied with “I + Se”. The HQ-I and HQ-Se coefficients were significantly lower than 1.0 for all tested treatments. I and Se could potentially harm the consumer if both the HQ coefficients were ≥1.0. The highest value of HQ-I and HQ-Se occurred after the application of a higher dose of “I + Se” (400 g I + 20 g Se ⋅ ha^–1^) and was 0.0074 for HQ-I and 0.00638 for HQ-Se.

**TABLE 5:** Percentage of Recommended Daily Allowance (RDA) for iodine (RDA-I) and selenium (RDA-Se) in 100-g portion of fresh carrot roots, I:Se molar ratio in fresh carrot roots, and hazard quotient (HQ) for intake of I and Se through the consumption of 100 g of fresh carrot roots by adults (70-kg body weight), depending on the foliar application of iodine and selenium.

	Storage roots				
Foliar application	Percent RDA-I in 100 g of fresh carrot roots (%)*	HQ-iodine for 100 g of fresh carrot roots	Percent RDA-Se in 100 g of fresh carrot roots (%)*	HQ-selenium for 100 g of fresh carrot roots	Molar mass ratio of I:Se in fresh carrot roots
Control	0.21 ± 0.03a	0.0003 ± 0.0001a	0.27 ± 0.03a	0.00033 ± 0.00003a	1.35:1 (±0.15:1)b
200 g I + 10 g Se	1.96 ± 0.30b	0.0030 ± 0.0005b	1.98 ± 0.19c	0.00283 ± 0.00026c	1.63:1 (±0.14:1)b
400 g I + 20 g Se	4.19 ± 0.58c	0.0074 ± 0.0016d	4.37 ± 0.45d	0.00638 ± 0.00082d	1.60:1 (±0.12:1)b
200 g I	1.95 ± 0.16b	0.0030 ± 0.0003b	0.26 ± 0.03a	0.00027 ± 0.00002a	13.50:1 (±1.23:1)c
400 g I	3.55 ± 0.44c	0.0053 ± 0.0007c	0.21 ± 0.02a	0.00027 ± 0.00002a	31.11:1 (±5.97:1)c
5 g Se	0.26 ± 0.07a	0.0004 ± 0.0001a	1.13 ± 0.12b	0.00150 ± 0.00011b	0.37:1 (±0.10:1)a
10 g Se	0.45 ± 0.15a	0.0007 ± 0.0003a	2.18 ± 0.12c	0.00319 ± 0.00024c	0.33:1 (±0.10:1)a
Test *F* for foliar application	*	*	*	*	*
Test *F* for foliar application × year of study	n.s.	n.s.	n.s.	n.s.	n.s.

*Application 200 g I + 10 g Se ⋅ ha^–1^ and 400 g I + 20 g Se ⋅ ha^–1^ by “I + Se” fertilizer, application of 200 g and 400 I ⋅ ha^–1^ by “Solo iodine” fertilizer, and application of 5 and 10 g Se ⋅ ha^–1^ by “Solo selenium”⋅fertilizer.*

Means followed by the same letters are not significantly different for *p* < 0.05; ±, standard error (*n* = 8). *Statistically significant; n.s., not significant for *p* < 0.05. The hazard to consumer exists when the value of HQ exceeds 1.0. Average for the years 2019–2020.

The ratio of I and Se in fresh carrot roots (converted to the molar mass of both elements) after applying the “I + Se” fertilizer in both doses was similar to the control (Table 5). After applying the fertilizer “Solo selenium” at both doses, the molar content ratio of I:Se in the roots was significantly lower than in the control. However, after application of “Solo iodine”, a significant extension of this ratio in favor of I was observed.

### Levels of Iodates, SA, BeA, and Their I Derivatives

When compared to the control treatment, the fertilizers “I + Se” and “Solo iodine” caused a decrease of 20% in the levels of BeA in the roots and increased the levels of 3,5-diISA by 20% in the leaves (Table 6). “I + Se” caused a significant increase in the levels of 2,3,5-triIBeA (3-fold) and I-Tyr (2-fold) and a significant decrease in BeA (1.7-fold) and 2-IBeA (3-fold) in the leaves. It caused a significant increase in IO_3_^–^ ions (5.2-fold) in the roots. The application of “Solo iodine” resulted in a significant increase in the levels of 5-ISA (2.2-fold), 2,3,5-triIBeA (12.0-fold), and I-Tyr (3.5-fold) and a 2-fold decrease in 4-IBeA and T3 in the leaves compared to that in the untreated control (Table 6). This fertilizer also caused a significant increase in the content of 5-ISA (1.9-fold), 4-IBeA (8.3-fold), 2,3,5-triIBeA (1.18-fold), T4 (1.9-fold), and I-Tyr (1.8-fold) in the roots. I and Se applied together as “I + Se” (200 g I + 10 g Se ⋅ ha^–1^), compared to I-alone “Solo iodine” (200 g I ⋅ ha^–1^), caused a significant decrease in the levels of 5-ISA (3.3-fold) 2-IBeA (1.27-fold), 2,3,5-triIBeA (3.8-fold), and I-Tyr (1.6-fold) in the leaves (Table 6). In the roots, it caused a significant decrease of 5-ISA (1.5-fold), 3,5-diISA (2.7-fold), 4-IBeA (2.5-fold), 2,3,5-triIBeA (1.26-fold), and T4 (1.4-fold).

**TABLE 6:** Content of iodates (IO_3_^–^), salicylic acid (SA), benzoic acid (BeA), iodosalicylates [5-iodosalicylic acid (5-ISA) and 3,5-diiodosalicylic acid (3,5-diISA)], iodobenzoates [2-iodobenzoic acid (2-IBeA), 4-iodobenzoic acid (4-IBeA), 2,3,5-triiodobenzoic acid (2,3,5-triIBeA)], iodotyrosine (I-Tyr), and plant-derived thyroid hormone analogs (PDTHAs), i.e., triiodothyronine (T3) and thyroxine (T4) in carrot storage roots–results only from 1 year of study.

Foliar application	Part of plants	(mg ⋅ kg^–^^1^ DW)					
		IO_3_^–^	SA	BeA	5-ISA	3,5-diISA	2-IBeA
Control	Leaves	0.0276 ± 0.008a	0.35 ± 0.032a	1.43 ± 0.22c	0.0018 ± 0.0003a	0.109 ± 0.015a	0.038 ± 0.002c
200 g I + 10 g Se		0.0231 ± 0.005a	0.37 ± 0.081a	0.84 ± 0.13a	0.0012 ± 0.0002a	0.129 ± 0.017b	0.011 ± 0.001a
200 g I		0.0402 ± 0.007a	0.32 ± 0.011a	1.22 ± 0,14bc	0.0040 ± 0.0003b	0.137 ± 0.012b	0.014 ± 0.004b
Test *F* for foliar application		n.s.	n.s.	*	*	*	*
Control	Roots	0.0062 ± 0.001ab	0.04 ± 0.005a	0.56 ± 0.07b	0.0038 ± 0.0007a	0.017 ± 0.003ab	0.015 ± 0.003a
200 g I + 10 g Se		0.0326 ± 0.016c	0.05 ± 0.004a	0.45 ± 0.07a	0.0047 ± 0.0006a	0.009 ± 0.001a	0.014 ± 0.001a
200 g I		0.0125 ± 0.008bc	0.07 ± 0.003a	0.44 ± 0.09a	0.0075 ± 0.0009b	0.025 ± 0.008b	0.015 ± 0.002a
Test *F* for foliar application		*	n.s.	*	*	*	n.s.

		**4-IBeA**	**2,3,5-triIBeA**	**T3**	**T4**	**I-Tyr**	

Control	Leaves	0.012 ± 0.002b	0.010 ± 0.002a	0.012 ± 0.004b	0.0070 ± 0.0002a	0.0031 ± 0.0004a	
200 g I + 10 g Se		0.019 ± 0.002b	0.031 ± 0.005b	0.011 ± 0.003b	0.0074 ± 0.0002a	0.0066 ± 0.0010b	
200 g I		0.005 ± 0.001a	0.120 ± 0.007c	0.006 ± 0.000a	0.0073 ± 0.0004a	0.0111 ± 0.0006c	
Test *F* for foliar application		*	*		n.s.	*	
Control	Roots	0.003 ± 0.001a	0.092 ± 0.007a	0.002 ± 0.001a	0.0106 ± 0.0011a	0.0036 ± 0.0014a	
200 g I + 10 g Se		0.010 ± 0.003b	0.086 ± 0.004a	0.011 ± 0.002b	0.0137 ± 0.0003a	0.0051 ± 0.0004ab	
200 g I		0.025 ± 0.002c	0.109 ± 0.007b	0.011 ± 0.003b	0.0202 ± 0.0026b	0.0065 ± 0.0008b	
Test *F* for foliar application		*	*	*	*	*	

Application 200 g I + 10 g Se ⋅ ha^–1^ and 400 g I + 20 g Se ⋅ ha^–1^ by “I + Se” fertilizer, and application of 200 g and 400 I ⋅ ha^–1^ by “Solo iodine” fertilizer. Means followed by the same letters separately for each part of plants are not significantly different for *p* < 0.05; ±, standard error (*n* = 4). *Statistically significant; n.s., not significant for *p* < 0.05. Results only from 2019.

None of the treatments resulted in statistically significant changes in levels of IO_3_^–^, SA, and T4 in the leaves and SA and 2-IBeA in the roots when compared to the control.

### Leaf and Root Dry Matter Content and Content of Total Dissolved Solids (Brix %), Sugars, and Carotenoids in Roots

The foliar application of “I + Se”, “Solo iodine” and “Solo selenium” (in all doses) had no significant effect on dry weight (Table 7), the Brix percentage, glucose, fructose, sucrose, total sugars, and carotenoids in carrots when compared to the control (Table 8). There were significant differences in the levels of total soluble solids between the treatments. The highest value of Brix (9.30%) was observed in carrot roots after the plant was sprayed with “I + Se” at a dosage of 400 g I + 20 g Se ⋅ ha^–1^. Alternatively, the highest concentration of all sugars was observed in the roots of plants sprayed with “Solo iodine” at a dose of 200 g I ⋅ ha^–1^ and then with “Solo selenium” at a dose of 20 g Se ⋅ ha^–1^, as well as with “I + Se” at a dose of 400 g I + 20 g Se ⋅ ha^–1^.

**TABLE 7:** Content of dry weight in leaves and storage roots of carrot, depending on the foliar application of iodine and selenium.

Foliar application	% D.W. in leaves	% D.W. in storage roots
Control	14.8 ± 0.79a	13.5 ± 0.84a
200 g I + 10 g Se	14.6 ± 0.60a	13.3 ± 0.84a
400 g I + 20 g Se	14.4 ± 0.65a	15.1 ± 1.76b
200 g I	14.6 ± 0.74a	13.4 ± 0.67a
400 g I	14.7 ± 0.72a	13.0 ± 0.58a
5 g Se	14.2 ± 0.57a	13.1 ± 0.62a
10 g Se	14.9 ± 0.86a	13.5 ± 0.65a
Test *F* for foliar application	n.s.	n.s.
Test *F* for foliar application × year of study	n.s.	n.s.

*Application 200 g I + 10 g Se ⋅ ha^–1^ and 400 g I + 20 g Se ⋅ ha^–1^ by “I + Se” fertilizer, application of 200 g and 400 I ⋅ ha^–1^ by “Solo iodine” fertilizer, and application 5 and 10 g Se ⋅ ha^–1^ by “Solo selenium”⋅fertilizer. Means followed by the same letters are not significantly different for *p* < 0.05; ±, standard error (*n* = 8); n.s., not significant for *p* < 0.05. Average for the years 2019–2020.*

**TABLE 8:** Content of and total soluble solids (Brix %); glucose, fructose, sucrose, and total sugars (the sum of the three sugars); and carotenoids in storage roots, depending on the foliar application of iodine and selenium.

Foliar application	Brix (%)^1^	Glucose (mg ⋅ 100 g^–^^1^ f.w.)	Fructose (mg ⋅ 100 g^–^^1^ f.w.)	Sucrose (mg ⋅ 100 g^–^^1^ f.w.)	Total sugars (mg ⋅ 100 g^–^^1^ f.w.)	Carotenoids (mg ⋅ 100 g^–^^1^ f.w.)
Control	9.15 ± 0.09abc	262.8 ± 32.2a	322.9 ± 121.0a	2,819.4 ± 536.4abc	3,405.0 ± 668.6ab	13.3 ± 0.59a
200 g I + 10 g Se	9.10 ± 0.07abc	297.3 ± 82.7ab	363.0 ± 130.8ab	2,736.0 ± 216.1ab	3,396.3 ± 319.5a	13.7 ± 0.39a
400 g I + 20 g Se	9.30 ± 0.04c	326.6 ± 84.1abc	400.2 ± 73.4bc	3,166.9 ± 350.6bc	3,893.7 ± 361.4bc	14.6 ± 0.31a
200 g I	9.05 ± 0.02ab	545.7 ± 79.1d	587.6 ± 210.6d	3,009.01674.8c	4,142.3 ± 159.3c	14.0 ± 0.76a
400 g I	9.10 ± 0.04abc	261.9 ± 49.4a	335.3 ± 82.4a	2,463.7 ± 776.7a	3,060.9 ± 820.8a	15.6 ± 1.30a
5 g Se	8.95 ± 0.13a	343.2 ± 21.8bc	405.7 ± 167.7bc	2,617.5 ± 846.7a	3,366.4 ± 1014.5a	14.6 ± 1.23a
10 g Se	9.25 ± 0.02bc	384.2 ± 92.8c	453.2 ± 57.4c	3,190.7 ± 485.1c	4,028.1 ± 459.8c	14.6 ± 0.79a
Test *F* for foliar application	*	*	*	*	*	n.s.
Test *F* for foliar application × year of study	–	n.s.	n.s.	n.s.	n.s.	n.s.

*1: Results of Brix and carotenoids are only from 2020, results of all sugars are average for the years 2019–2020.*

*Application 200 g I + 10 g Se ⋅ ha^–1^ and 400 g I + 20 g Se ⋅ ha^–1^ by “I + Se” fertilizer, application of 200 g and 400 I ⋅ ha^–1^ by “Solo iodine” fertilizer, and application 5 and 10 g Se ⋅ ha^–1^ by “Solo selenium”⋅fertilizer.*

Means followed by the same letters are not significantly different for *p* < 0.05; ±, standard error (*n* = 8). *Statistically significant; n.s., not significant for *p* < 0.05.

### Color of the Carrot Roots

Statistical analysis showed no differences in the *L*^∗^ parameter, i.e., the brightness of the color of the roots (Table 9). The values of the remaining parameters *a*^∗^, *b*^∗^, and *C*^∗^ showed significant differences, depending on the fertilizer used. The roots of the untreated carrot showed the lowest mean values for the color parameters *a*^∗^, *b*^∗^, and *C*^∗^. However, the highest values for these parameters were observed in carrots treated with the “Solo selenium” fertilizer, applied at a dose of 5 g Se ⋅ ha^–1^. There were no significant differences in the *a*^∗^, *b*^∗^, and *C*^∗^ color parameters for applications with higher and lower doses of “I + Se”, “Solo iodine” and “Solo selenium” fertilizers.

**TABLE 9:** Color parameters of carrot storage roots, depending on the foliar application of iodine and selenium–results only from 1 year of study.

Foliar application	L*	a*	b*	C*
Control	45.85a	27.34a	37.96a	46.78
200 g I + 10 g Se	46.95a	29.77c	39.89bc	49.77
400 g I + 20 g Se	46.16a	29.26c	39.77bc	49.37
200 g I	46.39a	27.66ab	38.53ab	47.42
400 g I	46.56a	28.69bc	39.57bc	48.88
5 g Se	46.96a	29.41c	40.68c	50.20
10 g Se	47.36a	28.59abc	38.76ab	48.16
Test *F* for foliar application	n.s.	*	*	n.c.

*Application 200 g I + 10 g Se ⋅ ha^–1^ and 400 g I + 20 g Se ⋅ ha^–1^ by “I + Se” fertilizer, application of 200 g and 400 I ⋅ ha^–1^ by “Solo iodine” fertilizer, and application 5 and 10 g Se ⋅ ha^–1^ by “Solo selenium”⋅fertilizer.*

Means followed by the same letters are not significantly different for *p* < 0.05; ±, standard error (*n* = 4). *Statistically significant; n.s., not significant for *p* < 0.05; n.c., this parameter is not statistically calculated. All results were obtained from the trials carried in 2020.

L* indicates color lightness (L* = 0 black, L* = 100 white).

a*, contribution of green (a* < 0) or red (a* > 0) color.

b*, contribution of blue (b* < 0) or yellow (b* > 0) color.

**FIGURE 2:**
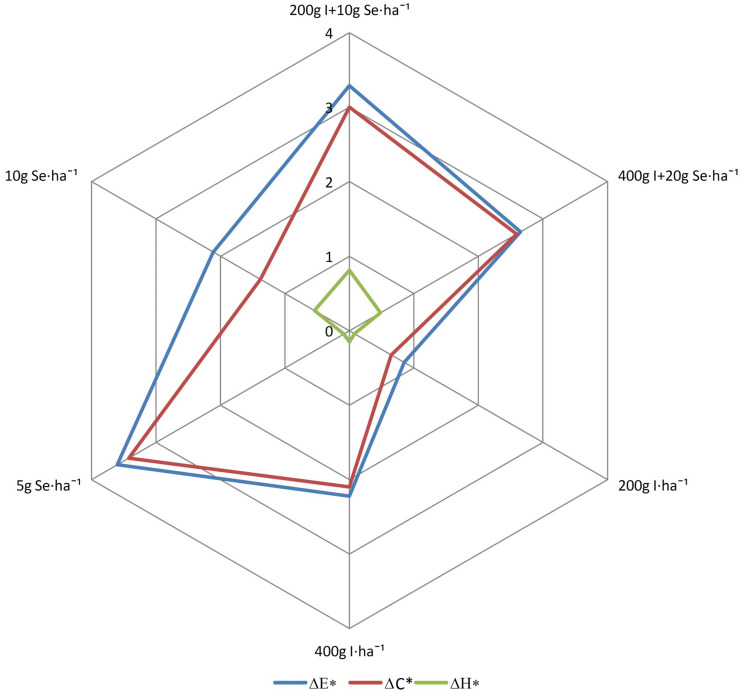
Effect of biofortification on color indices: Δ*E*^∗^, Δ*C*^∗^, Δ*H*^∗^. The mentioned parameters are calculated using the results of the average values of the basic parameters (*L*^∗^, *a*^∗^, *b*^∗^, *C*^∗^). In accordance to the guidelines of International Commission on Illumination, the parameter values of Δ*E*^∗^, Δ*C*^∗^, and Δ*H*^∗^ less than 2 indicate the lack of recognizable differences in the color by the observer; greater than 5 indicates the differences are clear and visible to any observer. Where Δ*E*^∗^, total color difference; Δ*C*^∗^, total saturation difference; ΔH^∗^, total hue difference. Application 200 g I + 10 g Se ⋅ ha^–1^ and 400 g I + 20 g Se ⋅ ha^–1^ by “I + Se” fertilizer, application of 200 g and 400 I ⋅ ha^–1^ by “Solo iodine” fertilizer, and application 5 g and 10 g Se ⋅ ha^–1^ by “Solo selenium”⋅fertilizer.

According to the guidelines of the CIE, a total color difference was not recognized for the roots of plants treated with the fertilizers “I + Se” at a dose of 400 g I + 20 g Se ⋅ ha^–1^ and “Solo iodine” at a dose of 200 g I ⋅ ha^–1^ (value Δ*E*^∗^ < 2) (Figure 2). For the other treatments, the parameter was between 2 and 5, which is a color difference that is recognized by a specialist. The total saturation difference (Δ*C*^∗^) for plants sprayed with “Solo iodine” at a dose of 200 g I ⋅ ha^–1^ had the lowest value. For this parameter, there were no differences visible to the eye in the roots of plants treated with “Solo selenium” (10 g Se ⋅ ha^–1^). No value greater than 2 was observed for total hue difference (Δ*H*^∗^) in any of the treatments, which is interpreted as no visible differences in the hue of the roots.

## Discussion

Simultaneous biofortification of crops with I and Se in agriculture is recommended because of the benefits of these elements for human and animal health, due to the deficiency of both elements in many regions of the world (Germ et al., 2015; Gonzali et al., 2017; Márquez et al., 2020). In addition, the combined application of I and Se in low doses can influence the plant positively. This effect can be observed in the crop yields and/or in the concentrations of nutrients, bioactive compounds, and nutraceuticals (Germ et al., 2015; Medrano-Macías et al., 2018).

Mao et al. (2014) conducted research with soil fertilization, using I and Se. They showed that the effectiveness of I and Se biofortification of wheat, maize, soybean, potato, canola, and cabbage was similar, regardless of whether the elements were applied independently or in combination. Foliar fertilization is considered more effective than soil fertilization for enriching plants with I and Se (Lawson et al., 2015).

The research presented in this article adds to the body of research currently being undertaken across the world. The effectiveness of enriching wheat and rice grains with these elements by foliar application was shown in experiments in several different countries (Zou et al., 2019; Cakmak et al., 2020). The effectiveness of foliar biofortification of I in plants depends on whether this element is applied alone or in combination with other minerals. Foliar application of a “cocktail” of three elements, I + Zn + Se resulted in a significant decrease in I accumulation in wheat grain, in comparison to the application of I alone (Cakmak et al., 2020). A similar decrease in the levels of I in wheat grains was observed with the application of a “cocktail” of I + Zn + Fe + Se, versus I alone (Zou et al., 2019). This research indicated that levels of Se were determined in the control plants and in the plants treated with the “cocktail,” but analysis for Se was not performed on plants treated with I. Golubkina et al. (2018) undertook similar studies on the Indian mustard and did not show a negative effect for the foliar application of I + Se (KI + Na_2_SeO_4_) on the accumulation of Se in plants when compared to the foliar application of Se alone (Na_2_SeO_4_). However, there was a noticeable decrease in the accumulation of I in the Indian mustard after the application of I + Se, compared to that in plants treated with I alone. The research is helpful in identifying the interactions for uptake and translocation of I and Se in various crops after foliar application of these elements, when applied “Solo” or in combination with other elements.

Foliar biofortification of plants with I and Se occurs because of transportation of these elements through the phloem, despite the relatively low mobility of I through phloem compared to more effective transportation through xylem. I and Se are translocated through the phloem from the leaves to other aerial parts of plants, including to grains or seeds (Cakmak et al., 2017) and fruits such as strawberries (Budke et al., 2020b), apples (Budke et al., 2020a), and tomatoes (Landini et al., 2011). Some authors indicate that despite the success of plants enrichment with I, redistribution of this element from the leaves to fruits, grains, or seeds by the phloem is limited (Muramatsu et al., 1995; Shinonaga et al., 2000; Salau et al., 2008). The enrichment of underground parts of plants, such as the roots of carrots or potato tubers with I or Se, is possible because of the transportation of these elements through the phloem from the leaves to underground tissues. This effect was demonstrated in studies on biofortification with I in the cultivation of carrots (Signore et al., 2018) and potatoes (Ledwożyw-Smoleń et al., 2020), as well as in research on biofortification with Se in carrots (Oliveira et al., 2018) and potatoes (Zhang et al., 2019). There are no data on whether the foliar application of I and Se simultaneously will enrich carrot roots. The research showed that both elements, applied as I^–^ and SeO_4_^2–^, were transported through the phloem from the leaves to the roots after foliar application. There was no interaction between the elements with respect to the level of the enrichment of the roots with I and Se. In case of the leaves, it was observed that combined application of I with Se (400 g I + 20 g Se ⋅ ha^–1^), compared to the application of I alone (400 g I ⋅ ha^–1^), had a synergistic effect on increasing the uptake and accumulation of I. However, no interaction was observed in the accumulation of Se in the leaves after foliar application of Se alone, and I + Se. The interaction between I and Se in foliar applications is most likely dependent on the plant species and the chemical form in which these elements are applied. In lettuce cultivation, foliar application of KIO_3_ had a synergistic effect on the uptake of SeO_4_^2–^, whereas Se in this form had no effect on the level of I accumulation in leaves after the plants were sprayed with KIO_3_ (Smoleń et al., 2014a).

Biofortification of I and Se in the roots of carrots does not have a negative effect on the yield of leaves and roots (total, marketable, and non-marketable yield); or on the dry weight in leaves and roots; or on the levels of total dissolved solids, sugars, and carotenoids in the carrot roots. Foliar application of I alone (200 g I ⋅ ha^–1^) caused a significant increase in sugar content in the roots. No plant damage was observed after foliar application of I, Se, and I + Se in the study. The study was not investigating ways to increase yield but rather to enhance the accumulation of I and Se in carrots. Similar effects were found by Smoleń et al. (2019) when soils were fertilized with I + Se (4 kg I ⋅ ha^–1^ as KI and 0.25 kg Se ⋅ ha^–1^ as Na_2_SeO_4_). There was no effect on yield, but enrichment of the four carrot varieties with I and Se was achieved.

Color assessment is one of the basic parameters assessed in the context of the quality of raw materials. It is the first and often decisive parameter in the choice of consumers. Instrumental assessment of color, thanks to the recommendations of the CIE, allows determining whether the color difference will be visible to the naked eye. In the presented research, it was found that the enrichment had a slight but statistically significant effect on the color of carrot roots. Gonnella et al. (2019) found no differences in the color of the leaves of different cabbage cultivars after the use of I fortification. Similar conclusions from the research were obtained by Montesano et al. (2016) and D’Imperio et al. (2016) for green peas and six species of leaf vegetables fortified with silicon compounds. Statistical differences in color after the use of Se fortification were also not found by Mimmo et al. (2017) study for strawberry fruit, although the results suggest that these differences should be visible to the naked eye. In the case of the cited data, the color parameters of the tested raw materials depend mainly on the content of chlorophylls and polyphenolic compounds, which do not always change under the influence of fortification (Smoleń et al., 2014a). Carotenoids determine the color of carrot roots. In the literature, information on the effect of I and Se biofortification on the content of these compounds in carrot roots could be found (Vanuze Costa de Oliveira et al. Oliveira et al., 2018; Smoleń et al., 2019). The results of the carotenoids content obtained in the experiment were not statistically significant what they comply with cited literature.

The results of this study show that the doses and types of foliar fertilizers (“I + Se”, “Solo iodine” and “Solo selenium”) allowed for enrichment of carrot roots with I and Se safely during cultivation. The plants were physiologically able to tolerate the doses of I and Se applied. Furthermore, the chemical form of I^–^ and Se (SeO_4_^2–^), when combined with an organic stabilizer/enhancer^®^, allowed for a higher uptake of I and Se by the epidermis of leaves and better transport through the phloem to the roots. Evidence was also provided that the foliar application of I in the form of I^–^ ions stabilized by organic stabilizer/enhancer^®^ allowed for uptake, transport, and metabolization in the whole carrot plant. The results of the analysis in this study showed the levels of I, ions I^–^, and organic metabolites of I compounds in leaves and roots. The dominant form of I in the roots and leaves were iodides (IO_3_^–^), which were taken up from the soil. On average, I^–^ represented 77.4% of total level of I in the roots for treatments with fertilizers “I + Se” and “Solo iodine”, and 50.1% for the untreated control and for plants treated with “Solo selenium”. On average, I^–^ represented 55.5% of the total content of I in the leaves for plants treated “I + Se” and “Solo iodine” and 63.6% for the control and “Solo selenium” treatment.

These results demonstrate the effective translocation of I^–^ ions from the leaves to the roots of carrot plants. Chemical analysis also showed that iodides were utilized in carrot plants in metabolic pathways for the synthesis of iodosalicylates, iodobenzoates, I-Tyr, and PDTHA. Similar results were found for lettuce plants (Smoleń et al., 2020). I can also be incorporated into other groups of compounds, such as polysaccharides, lipids, polyphenols, and proteins (Millard, 1988; Hou, 2009).

As already mentioned, no significant differences were found in the levels of total I accumulation in the leaves and roots when doses of 200 g I + 10 g Se ⋅ ha^–1^ were applied as “I + Se” versus 200 g I ⋅ ha^–1^ as “Solo iodine”. However, significant interactions between I and Se in plants were observed for the levels of I metabolites in carrots. The results showed that combined applications of I + Se, opposed to the use of I alone, had significant impacts at a biochemical level. First, a decreasing synthesis/accumulation of 5-ISA, 2-IBeA, 2,3,5-triIBeA, and I-Tyr and an increasing synthesis/accumulation of 4-IBeA, T3 were found in the leaves. Second, a decreasing synthesis/accumulation of 5-ISA, 3,5-diISA, 4-IBeA, 2,3,5-triIBeA, and T4 was found in the roots. One of the most important findings of the study was that the organic metabolites of I, namely I-Tyr, 5-ISA, and 2,3,5-triIBeA, were synthesized and accumulated in higher levels in the carrot plant after foliar application of I. While these compounds were found in plants not treated with I in studies on tomatoes (Halka et al., 2019) and lettuce plants (Smoleń et al., 2020), fertilization with I increased their levels in these plants (Halka et al., 2019; Smoleń et al., 2020), which was also confirmed in this study.

It is noteworthy that the synthesis of two of iodosalicylates (5-ISA 3,5-diISA) decreased in carrots after the combined application of I and Se, compared to when I alone was applied. These are PDTHA precursors in plants (Smoleń et al., 2020). However, the application of I alone in contrast to the application of I and Se together caused a significant reduction of the content of T3 (but not T4) and simultaneously increased the content of I-Tyr in carrot leaves, compared to the control. I-Tyr is an I amino acid that most likely acts to store I in the plant. This amino acid also participates in the complex mechanism of PDTHA synthesis (Smoleń et al., 2020). The physiological and biochemical functions and range of actions of PDTHA in plants have not yet been described in the literature. There are studies that show that the proteins T3 and T4 synthesized in plants are homologous to human proteins (Eneqvist et al., 2003). The T3 and T4 proteins that occur in cereals, tomatoes, potatoes and in *Arabidopsis thaliana* are more closely related to the protein present in the *Homo sapiens* organism than to those in bacteria and fungi. In *A. thaliana*, exogenous T4 is bound by the transthyretin-like protein (Pessoa et al., 2010). In the human body, transthyretin-like proteins belong to the group of proteins that include T4-binding globulin and albumin (Power et al., 2000). Based on this, it can be assumed that PDTHA may have a regulatory function in plants.

5-iodosalicylic acid is the primary iodosalicylate in plants and one of the three metabolites most synthesized in carrot plants after foliar application of I (Smoleń et al., 2020). The results indicate that 5-ISA was transported from the leaves to the roots of carrot plants, where it was converted to 3,5-diISA. The direction of transformations of 5-ISA to 3.5-diISA is consistent with reports in the literature (Halka et al., 2019; Smoleń et al., 2020).

An important issue is an increase of the synthesis of 2,3,5-triIBeA, subsequent to the application of fertilizers containing I. In the literature, 2,3,5-triIBeA is regarded as an auxin inhibitor (Saniewski et al., 2014). Despite the fact that the levels of 2,3,5-triIBeA (especially in leaves) increased after foliar application of I, compared to the control, this did not have a negative effect on the functioning of auxins in carrots. However, there is a lack of influence related to the effect of fertilizers containing I (“I + Se” and “Solo iodine”) on the yield of carrot roots and leaves.

The application of trace elements with organic stabilizers is an acceptable plant biofortification strategy in the cultivation of plants. The stabilizer/enhancer^®^ contained in all three fertilizers used in this study is based on organic compounds naturally present in plants, which makes it safe for the plants and consumers. For example, Dávila Rangel et al. (2020) tested the application of chitosan-I complex (Cs-KIO_3_, Ch-KI) to enrich lettuce with I successfully.

The optimal molar ratio of I:Se for the daily intake by the consumer is within the range of 4.4–8.8:1. This range is based on the RDA-I and RDA-Se for consumers in various age groups, namely, children, adolescents, adults, and breastfeeding mothers (Institute of Medicine, 2000; Andersson et al., 2007). Therefore, plant biofortification protocols should aim to enrich plants with I and Se to obtain a molar ratio for both elements in the range of 4.4–8.8:1. The upper levels of biofortification of the edible parts of crop plants must be safe for the consumer, indicating the need for values of I and Se of HQ < 1.0.

It is not easy to achieve optimum ratios of I:Se in cereal yields when simultaneously enriching with I and Se. Research by Cakmak et al. (2020) in wheat cultivation showed that foliar application with 2.3 mM I + 15.9 mM zinc + 0.05 mM Se (molar ratio of I:Se = 46:1) resulted in grains with a molar ratio of I:Se = 0.23:1, whereas application of I alone resulted in a molar ratio of I:Se in grains of 4.35:1. Zou et al. (2019) enriched wheat grains with both elements using foliar application of I + Se in a ratio of 50:1, but the resultant molar ratio of I:Se in grains was 0.45:1. Mao et al. (2014) applied fertilizers with a mixture of I + Ze + Se in a ratio of I:Se 2.8:1. The result was enrichment of plants with I and Se, achieving a molar ratio of I:Se for wheat and maize grains of 0.0027:1 and 0.0067:1, respectively; of 0.021:1 for potato tubers; and of 0.037:1 for cabbages. Similarly, as in the case of the cereals, potatoes, and cabbages mentioned above (Mao et al., 2014; Zou et al., 2019; Cakmak et al., 2020), the molar ratio of I:Se in fertilizers did not translate into similar molar ratios of I:Se in the roots. Smoleń et al. (2019) applied pre-sowing fertilization with I and Se in the ratio of 9.9:1 for four carrot varieties. Biofortification was obtained, but the molar ratio of I:Se in the roots was 0.24:1. This resulted from better absorption of I than Se in the soil and differences in the rate of uptake and transport from the roots to leaves, as well as metabolism in plants (Smoleń et al., 2019). A better ratio of I:Se biofortification in the roots of carrots was achieved when applying pre-sowing and top dressing in soil fertilization (Smoleń et al., 2016a), rather than pre-sowing application only (Smoleń et al., 2019).

The molar mass ratio of I:Se in the “I + Se” fertilizer is 12.4:1. Foliar application of this fertilizer allowed for molar concentrations of approximately 1.6:1 of I:Se in the roots. The ratio of the molar content deviated from the optimal level, resulting from the values of RDA-I and RDA-Se. Despite the significantly increased accumulation of I and Se, the foliar application of “I + Se” fertilizer allowed for an I:Se ratio at a similar level to the one observed in the control plants. Therefore, it can be concluded that the metabolic processes of I and Se in carrots after foliar application of “I + Se” allowed for maintenance of the equilibrium state of biofortification with I:Se, as was observed in the control plants. We assumed that there were mechanisms for self-regulation of the physiological concentration (accumulation) of I and Se, which is based on the methylation process of I in the plants (Itoh et al., 2009; Izydorczyk et al., 2020) and Se (Kápolna et al., 2009; Sors et al., 2009).

## Conclusion

The effect of biofortification of carrot roots with I and Se after foliar application of the fertilizers “I + Se” (I-Se fertilizer), “Solo iodine” and “Solo selenium” was studied. The foliar biofortification strategy had no negative impact on the yield of leaf biomass and roots (total and marketable yield) in carrot plants or the nutritional value of carrots. Elements applied as foliar applications had no influence on the content of total dissolved solids, sugars, and carotenoids in roots or on dry weight of roots and leaves. The fluctuations in the color of the roots were negligible and not noticeable to the human eye.

The results proved that foliar application is an effective way to biofortify roots with I and Se, which were transported from the leaves to the roots, probably through the phloem. No interaction (synergistic or antagonistic) was identified between the applied elements with regard to the effectiveness of biofortification of roots in any of the treatments.

The accumulation level of I metabolites in leaves and roots when compared to the control, in the light of applications of I + Se, is important from the perspective of plant biochemistry and physiology. This provides information about plant nutrition and I metabolism in carrot plants. The levels of organic I metabolites and PDTHA content in roots can be considered safe for consumers as the value of the HQ-I index was lower than 1.0, and the percentage of the RDA-I in 100 g of fresh carrot roots was a maximum of 4.16% (for treatment with 400 g I + 20 g Se ⋅ ha^–1^). The HQ-Se values also indicated that the Se-enriched carrot was safe for the consumer. The percentage of RDA-Se in 100 g of fresh carrot roots was a maximum of 4.37% in the combination treatment with 400 g I + 20 g Se ⋅ ha^–1^. This study has allowed for the development of foliar spraying protocols for the biofortification of carrot plants with I and Se.

## Data Availability Statement

The original contributions presented in the study are included in the article/Supplementary Material, further inquiries can be directed to the corresponding author/s.

## Author Contributions

RR-L, MG, KA, HK, and SS conceived and designed the field experiments. SS and MG conducted the field experiments and wrote the manuscript, together with RR-L and KA. JP, ŁS, and ML-S conducted the analytical investigations. SS and RR-L supervised the analytical investigations. SS provided resources to conduct the field experiments and analytical investigations. SS and ŁS analyzed and supervised the statistical data analysis. All authors contributed to manuscript revision, reading, and approved the submitted version.

## Conflict of Interest

HK is CEO of Intermag. KA is Director of R&D at Intermag. RR-L and MG are employees of Intermag Sp. z o.o., Olkusz, Poland, a company active in the sector of fertilizers and biostimulants. The remaining authors declare that the research was conducted in the absence of any commercial or financial relationships that could be construed as a potential conflict of interest.

## Abbreviations

I-Tyr: iodotyrosine
PDTHA: plant-derived thyroid hormone analogs
RDA: Recommended Daily Allowance
RDA-I: Recommended Daily Allowance for iodine
RDA-Se: Recommended Daily Allowance for selenium
HQ: hazard quotient
SA: salicylic acid
BeA: benzoic acid
5-ISA: 5-iodosalicylic acid
3,5-diISA: 3,5-diiodosalicylic acid
2-IBeA: 2-iodobenzoic acid
4-IBeA: 4-iodobenzoic acid
2,3,5-triIBeA: 2,3,5-triiodobenzoic acid
T3: triiodothyronine
T4: thyroxine
D.W.: dry weight
F.W.: fresh weight.
