# Supplementary material for: Effectiveness of Foliar Biofortification of Carrot With Iodine and Selenium in a Field Condition
Source: Front Plant Sci. 2021 May 21;12:656283. doi: 10.3389/fpls.2021.656283 (PMC8177008; doi:10.3389/fpls.2021.656283)
Supplement: Supplementary file 1 [file Table_1.docx]

Table S1. Total selenium content in carrot leaves and storage roots depending on the foliar application of I and Se – results from the screening test carried out in the greenhouse.

| Foliar  application | Selenium content | | |
| --- | --- | --- | --- |
|  | Roots  (µg∙kg^-1^ DWM) | Leaves  (µg∙kg^-1^ DWM) |  |
| Control | 13.7±3.9a | 26.5±8.1a |  |
| 100 g I + 5 g Se | 91.8±13.9b | 380,7±51,1b |  |
| 200 g I + 10 g Se | 113.6±12.3c | 411.5±69.8b |  |
| 400 g I + 20 g Se | 162.7±16.5d | 502.2±56.0c |  |
| 5 g Se | 106.6±13.6bc | 388,5±51.8b |  |
| 10 g Se | 157,2±9,6d | 506,1±46.2c |  |
| 20 g Se | 152.3±14.6d | 579,9±49,8d |  |
| 100 g I | 20.2±3.8a | 17.7±9.2a |  |
| 200 g I | 17.8±2.1a | 25.7±10.9a |  |
| 400 g I | 21.9±3.5a | 16.4±5.0a |  |
| Test *F* | * | * |  |

Means followed by the same letters are not significantly different for p < 0.05; „±” – standard error (n=4); * ˗ statistically significant for P < 0.05.
